# Vertebral Compression Fractures: Factors Predicting Failure of Vertebral Augmentation Kyphoplasty

**DOI:** 10.1055/s-0046-1817022

**Published:** 2026-03-25

**Authors:** Joana Araújo de Azevedo, Vasco Campos, Paulo Gil Ribeiro, Nuno Oliveira, Pedro Varanda, Bruno Direito-Santos

**Affiliations:** 1 Department of Orthopedic Surgery, Hospital de Braga, Braga, Portugal; 2 School of Medicine, Universidade do Minho, Braga, Portugal

**Keywords:** bone cements, fractures, compression, kyphoplasty, spine, cifoplastia, cimento ósseo, coluna vertebral, fraturas por compressão

## Abstract

**Objective:**

To identify predictors of kyphoplasty failure in patients with vertebral compression fractures (VCFs), which may assist clinical decision-making and optimize therapeutic outcomes.

**Methods:**

The present retrospective cohort study included patients with VCFs treated by kyphoplasty, categorized based on the presence or absence of treatment failure. Forty records were evaluated with a minimum follow-up time of 6-months, and 6 cases were excluded because they did not meet the inclusion criteria. Variables analyzed included age, sex, time to surgery, fracture level, AO Spine Injury Classification, and osteoporotic fractures (OF) score. Radiological parameters such as vertebral body height, regional and segmental kyphotic angles were measured pre- and postoperatively. Additionally, we assessed the proportion of vertebral body occupied by cement, with emphasis on its anterior and posterior distribution.

**Results:**

Among 34 patients, 20 (58.8%) had successful outcomes, meaning absence of failure criteria until the last follow-up (group 1), while 14 (41.2%) experienced failure (group 2). Failure was defined based on the presence of adjacent vertebral fracture, vertebral re-collapse, or recurrence of incapacitating pain. Kyphoplasty led to significant improvements in vertebral body height and kyphotic angles. In regression analysis, only the posterior cement distribution percentage emerged as an independent predictor of failure (adjusted odds ratio [OR] = 1.684; *p* = 0.012). Increased posterior vertebral height gain showed a trend toward failure, whereas anterior height gain appeared protective.

**Conclusions:**

A higher percentage of cement located posteriorly within the vertebral body is associated with increased kyphoplasty failure risk. These findings highlight the importance of cement distribution patterns in surgical planning for VCFs.

## Resumo

**Objetivo:**

Identificar fatores preditivos de falha da cifoplastia em pacientes com fraturas por compressão vertebral (FCV), o que pode auxiliar na tomada de decisões clínicas e otimizar os resultados terapêuticos.

**Métodos:**

Este estudo de coorte retrospectiva incluiu pacientes com FCV submetidos à cifoplastia e categorizados com base na presença ou ausência de falha do tratamento. Foram avaliados 40 prontuários com tempo mínimo de acompanhamento de 6 meses; 6 casos foram excluídos por não atenderem aos critérios de inclusão. As variáveis analisadas foram idade, sexo, tempo até a cirurgia, nível da fratura, pontuação do Sistema de Classificação de Lesões da Coluna Vertebral da AO e escore de fraturas osteoporóticas (OF). Parâmetros radiológicos, como altura do corpo vertebral e ângulos cifóticos regionais e segmentares, foram medidos antes e após a cirurgia. Além disso, a proporção do corpo vertebral ocupada por cimento foi analisada, com ênfase em sua distribuição anterior e posterior.

**Resultados:**

Dentre os 34 pacientes, 20 (58,8%) apresentaram resultados satisfatórios, ou seja, ausência de critérios de falha até o último acompanhamento (grupo 1), enquanto 14 (41,2%) apresentaram falha (grupo 2). A falha foi definida pela presença de fratura vertebral adjacente, colapso vertebral recorrente ou recidiva de dor incapacitante. A cifoplastia gerou melhora significativa na altura do corpo vertebral e nos ângulos cifóticos. À análise de regressão, apenas a porcentagem de distribuição posterior do cimento emergiu como fator preditivo independente de falha (razão de chances ajustada = 1,684; *p* = 0,012). O aumento do ganho de altura vertebral posterior indicou tendência à falha, enquanto o ganho de altura anterior pareceu ter efeito protetor.

**Conclusão:**

A maior porcentagem de cimento localizada posteriormente no corpo vertebral está associada a um aumento do risco de falha da cifoplastia. Esses achados destacam a importância dos padrões de distribuição do cimento no planejamento cirúrgico de FCVs.

## Introduction

Vertebral compression fractures (VCFs) are a relatively frequent condition, in most cases because of osteoporosis. The collapsed vertebrae is a cause of acute and chronic pain, limiting mobility and declining quality of life. 1 These fractures may be caused by low-energy trauma or even sudden movements or efforts. Radiographs usually show diminished vertebral body height, decreased radiodensity, anterior wedging, and, less frequently, involvement of the posterior vertebral wall. Computed tomography (CT) and magnetic resonance imaging (MRI) play complementary roles in assessing the extent of fractures and identifying any neurological complications. 2 Conservative treatment is the first option, through pain medications, use of orthopedic braces, physical therapy, and lifestyle modifications. In case of persistent pain or functional limitations, more invasive interventions may be necessary.

Kyphoplasty is a minimally invasive surgical technique designed specifically for the treatment of VCFs. Vertebroplasty is another elective alternative. Kyphoplasty was introduced to overcome some limitations of vertebroplasty, attempting to reduce the fracture before cement injection. It involves a series of steps aimed at restoring vertebral heights, reducing kyphotic angles, achieving pain relief, but also preventing subsequent fractures and further deformity of the spine, which can lead to long-term complications such as kyphosis. Some recent literature has suggested that vertebroplasty and kyphoplasty are equivalent in providing pain relief and improved function. 3 The decision to perform these interventions must be cautiously considered and contemplate the risks and benefits. 4 5 Previous studies have identified factors such as steroid use, fracture at the thoracolumbar junction level, low bone mineral density (BMD), and cement leakage as possible factors that can influence outcomes after kyphoplasty. 6 7 8 However, many relevant variables were not assessed, like cement distribution, differential increase in posterior and anterior heights, among others, highlighting the need for further study of potential predictors of kyphoplasty failure.

Our aim was to evaluate kyphoplasty outcomes and explore potential predictors of treatment failure, through evaluation of demographic factors and surgery related parameters, to optimize the surgical procedure and its results.

## Materials and Methods

Ethics approval and consent were granted by the institution's ethics committee under number 130/2024.

We have proceeded to a retrospective, cohort, longitudinal, unicentric and analytical study, including all patients who underwent percutaneous vertebral kyphoplasty as treatment for VCFs at our hospital between 2017 and 2024. Percutaneous bilateral balloon kyphoplasty was performed in all patients. Many surgeons participated in the current study, resulting in technical variability that could hamper the results, and the amount of cement injected in each case was impossible to determine, but a mean of 2 to 4 cc was used in all cases. Inclusion criteria were history of osteoporotic VCF treated with kyphoplasty, age ≥ 18 years old, clinical/imaging follow-up period longer than 6 months. We have excluded all patients with pathological fractures and neurological deficits.

### Data Collection

Clinical and demographic data were collected from patient records, such as age, sex, associated comorbidities, level of fracture, type of fracture (AO Spine Injury Classification System), and classification according to the osteoporotic fractures (OF) score, timing to surgery, follow-up time, and postoperative complications. 9 Failure was considered when limitative pain for daily activities, adjacent vertebral fracture, or vertebral re-collapse was identified.

**Fig. 1:**
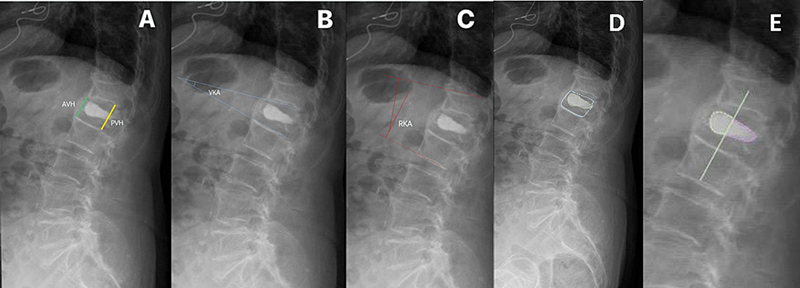
AVH/PVH/VKA/RKA and cement distribution measurement protocol. **Abbreviations** : AVH, anterior vertebral heigh; PVH, posterior vertebral height; RKA, regional kyphotic angle; VKA, vertebral kyphotic angle. ( **A** ) Measurement protocol for AVH (green line) and PVH (yellow line); ( **B** ) measurement protocol for VKA (blue lines); ( **C** ) measurement protocol for RK using Cobb's angle (red lines); ( **D** ) measurement of the vertebral area; and ( **E** ) measurement of the anterior cement area.

The radiological parameters measured pre- and postsurgically were anterior and posterior vertebral body height, vertebral kyphotic angle (VKA), and regional kyphotic angle/Cobb angle (RKA) of the fractured segment 10 ( Fig. 1 ).

Additionally, cement distribution was assessed by measuring the percentage of the vertebral area occupied by cement (% vertebral area with cement), the percentage of total cement in the anterior region of the vertebral body (% anterior cement), and the percentage of the total cement in the posterior region of the vertebra (% posterior cement) ( Fig. 1 ). This variable was measured through a lateral radiography, delimitating the cement mass inside the vertebral body using the Sectra software (Sectra AB), and with the percentage values being used to normalize the measurement.

### Statistical Analysis

Data was analyzed with the IBM SPSS Statistics for Windows (IBM Corp.) software, version 29.0.

Comparisons between pre- and postsurgery vertebral heights and angles were performed with the t-Student paired samples test, or Willcoxon test if normality was not assured.

Comparisons between quantitative variables, such as age, vertebral heights and angles, cement distribution and time to surgery, with procedure failure were performed with the t-Student independent samples or Mann-Whitney test for non-normal variables. To analyze the association between failure and the categorical variables, such as AO and OF scores and level of fracture, the Chi-squared test was used. The Fisher's exact test was used as an alternative to Chi-squared tests when Cochran's rules were not met.

A multivariate logistic regression was employed to analyze the contribution of all potential predictor variables and the occurrence of kyphoplasty failure. The variables included in the regression were the ones that had a *p* -value < 0.20. This allowed for the creation of a model, using these variables, that could partially explain the outcome.

For all tests, significance was considered for *p* -values < 0.05.

## Results

Thirty-four patients met the criteria and were enrolled in the present study. Among them, 20 cases (58.8%) were included in group 1, meaning no failure was observed, and group 2 had 14 cases (41.2%) of complications ( Table 1 ).

**Table 1:** Characteristics of groups 1 and 2

	Group 1	Group 2
**Number of patients**	20	14
**Age (years)**	70.95 ± 13.59	72.71 ± 9.05
**Gender**		
**Male**	5 (25%)	5 (35.71%)
**Female**	15 (75%)	9 (64.29%)
**Fractured segment**		
**D6**	1 (5%)	0
**D9**	1 (5%)	0
**D10**	1 (5%)	2 (14.3%)
**D11**	3 (15%)	1 (7.1%)
**D12**	4 (20%)	3 (21.4%)
**L1**	3 (15%)	4 (28.6%)
**L2**	2 (10%)	0
**L3**	5 (25%)	3 (21.4%)
**L4**	0	1 (7.1%)
**Time from injury to surgery (months)**	6 (5)	6 (6)
**AVH-pre (cm)**	1.72 ± 0.49	1.87 ± 0.62
**PVH-pre (cm)**	2.64 ± 0.51	2.61 ± 0.58
**VKA-pre (degrees)**	13.71 ± 7.3	11.36 ± 6.36
**RKA-pre (degrees)**	18.23 ± 6.41	19.21 ± 6.42
**AVH-post (cm)**	2.07 ± 0.39	2.07 ± 0.6
**PVH-post (cm)**	2.84 ± 0.464	2.95 ± 0.39
**VKA-post (degrees)**	10.04 ± 6.7	11.29 ± 6.39
**RKA-post (degrees)**	16.39 ± 15.16	18.47 ± 9.11
% AVHB ([post–pre]*100)/pre)	20.85 (22.29)	12.32(12.35)
%PVHB ([post–pre]*100)/pre)	2.36 (18.61)	9.843 (23.29)
**Dif RKA (post–pre) (degrees)**	1.75 (4.15)	1.55 (2.37)
**%Vertebral area with cement**	44.34 ± 10.20	44.93 ± 10.43
**%Anterior cement**	69.25 (10.52)	48.80 (9.61)
**%Posterior cement**	38.14 ± 6.85	51.57 ± 6.66
**AO SPINE**		
**A1**	18 (90%)	13 (91.2%)
**A2**	1(5%)	1 (2.9%)
**A3**	1(5%)	2 (5.9%
**OF score**		
**OF1**	1 (5%)	0
**OF2**	16 (80%)	10 (71.4%)
**OF3**	2 (10%)	5 (14.7%)
**OF4**	1(5%)	2 (5.9%)

**Abbreviations:** AVH, anterior vertebral height; PVH, posterior vertebral height; RKA, regional kyphotic angle; VKA, vertebral kyphotic angle.

**Note:** The results are expressed as n (%), mean ± standard deviation, or median (IQR).

Incapacitating pain was the most frequent cause of procedure failure (35.3%). Table 2 shows the prevalence of each cause.

**Table 2:** Frequencies of types of postprocedural failure

Type of postprocedural failure	
None	20 (58.8%)
Incapacitating pain	12 (35.3%)
Vertebral recollapse	1 (2.9%)
Adjacent vertebral fracture	1 (2.9%)
Cement leakage	0

When comparing the overall values before and after the procedure, posterior vertebral height (PVH) ( *p* < 0.001), VKA ( *p* < 0.001), anterior vertebral height ( *p* < 0.001) and regional kyphotic angle ( *p* = 0.003) showed to be significantly different between the 2 assessments, with a tendency to an increase in PVH and AVH, as well as a decrease in the VKA and RKA after the surgery, supporting the validity of kyphoplasty as a method for restoring normal vertebral body height and morphology. Further statistical analysis is presented in Table 3 .

**Table 3:** Assessment of variables before and after surgical intervention

	Pre	Post	*p*	Effect size
PVH M (SD)	2.63 (0.53)	2.89 (0.43)	**< 0.001**	*d =* 0.54
VKA M (SD)	12.74 (6.93)	10.55 (6.5)	**<0.001**	*d =* −0.44
AVH Mdn (IQR)	1.74 (0.64)	2.02 (0.67)	**< 0.001**	*r =* 0.56
RKA Mdn (IQR)	19.06 (13.2)	17.25 (14.3)	**0.003**	*r =* −0.36

**Abbreviations:** AVH, anterior vertebral height; IQR, interquartile range; M, mean; Mdn, median; PVH, posterior vertebral height; RKA, regional kyphotic angle; VKA, vertebral kyphotic angle.

We did not find a statistically significant relation between gender, OF score, AO Spine Classification, and fracture level with procedural failure. However, in the failure group, a higher percentage of male patients (35.7%) was observed compared to that of group 1 (25.0%), despite not being statistically significant ( *p* = 0.704). Regarding the OF score, the most frequent classification on both groups was OF 2 (80% in group 1 and 71.4% in group 2).

The most frequent type of fracture was in both groups was A1, and lumbar fractures were more prevalent in the failure group (57.1%), but no statistically significant differences were found. The results are shown in Table 4 .

**Table 4:** Association between the categorical variables in this study and procedural failure

	No failure	Failure	*p* -value	Effect size
**Gender***n* (%)			0.704	Φ = −0.12
Male	5 (25.0)	5 (35.7)		
Female	15 (75.0)	9 (64.3)		
**OF score***n* (%)			0.889	Φ _c_ = 0.215
OFS1	1 (5.0)	0		
OFS 2	16 (80.0)	10 (71.4)		
OFS 3	2 (10)	3 (21.4)		
OFS 4	1 (5.0)	1 (5.0)		
**AO spine***n* (%)			1	Φ _c_ = 0.151
A1	18 (90.0)	13 (92.9)		
A2	1 (5.0)	0		
A3	1 (5.0)	1 (7.1)		
**Fracture level***n* (%)			0.738	Φ = −0.70
Thoracic	10 (50.0)	6 (42.9)		
Lumbar	10 (50.0)	8 (57.1)		

**Abbreviations:** AO, AO Spine Injury Classification System; OF, osteoporotic fractures.

**Notes:** The results are expressed as n (%), mean ± standard deviation, or median (IQR). n: absolute frequency. Φ Phi (effect size), Φc (Cramer's V).

When comparing radiological variables with failure, we found that the failure group showed a statistically significant higher percentage of cement distribution on the posterior regions of the vertebra (51.7% vs. 38.1%; *p* = 0.001) and a smaller percentage of cement in the anterior half of the vertebra (48.8% vs 69.3%; *p* = 0.001). The rest of the variables showed no statistically significant differences; however, the % of PVH variation showed to be marginally significant ( *p* = 0.163).

The results are shown in Table 5 .

**Table 5:** Association between the variables studied and treatment failure

	No failure (n = 20)	Failure (n = 14)	*p* -value	Effect size
Age M (SD)	70.95 (13.59)	72.71(9.05)	0.675	*d = 0.15*
Time from injury to surgery (months) Mdn (IQR)	6 (5)	6 (6)	0.958	*r* = 0.16
AVH-pre (cm) M (SD)	1.72 ± 0.11	1.87 ± 0.17	0.449	*d = 1.05*
PVH-pre (cm) M (SD)	2.64 ± 0.11	2.60 ± 0.16	0.861	*d = 0.36*
VKA-pre (degrees) M (SD)	13.71 ± 1.63	11.36 ± 1.70	0.340	*d = 1.41*
RKA-pre (degrees) M (SD)	18.23 ± 3.78	19.21 ± 1.72	0.813	*d = 0.33*
AVH-post (cm) M (SD)	2.08 ± 0.09	2.07 ± 0.16	0.984	*d = 0.07*
PVH-post (cm) M (SD)	2.84 ± 0.10	2.95 ± 0.11	0.462	*d = 1.05*
VKA-post (degrees) M (SD)	10.04 ± 1.49	11.29 ± 1.71	0.589	*d = 0.78*
RKA-post (degrees) M (SD)	16.39 ± 3.39	18.47 ± 2.44	0.650	*d = 0.7*
% AVH variation Mdn (IQR)	20.85 (22.29)	12.32 (12.35)	0.221	*r* = 0.21
%PVH variation M (SD)	2.36 (18.61)	9.84 (23.29)	0.163	*d = 0.35*
Dif RKA (degrees) Mdn (IQR)	1.75 (4.15)	1.55 (2.37)	0.587	*r* = 0.09
%Vertebral area with cement M (SD)	44.34 ± 10.20	44.93 ± 10.43	0.869	*d =* 0.06
%Anterior cement Mdn (IQR)	69.25 (10.52)	48.80 (9.61)	**0.001**	*r* = −0.80
%Posterior cement M (SD)	38.14 ± 6.85	51.57 ± 6.66	**0.001**	*d* = 6.77

**Abbreviations:** AVH, anterior vertebral height; IQR, interquartile range; M, mean; Mdn, median; PVH, posterior vertebral height; RKA, regional kyphotic angle; SD, standard deviation; VKA, vertebral kyphotic angle.

**Note:** The results are expressed as n (%), mean ± standard deviation, or median (IQR).

### Logistic Regression Models

Finally, a logistic regression model was performed. Each of the variables that showed statistically significant differences according to kyphoplasty failure and the variable that showed a *p* -value < 0.20 were included. Therefore, %anterior cement, %posterior cement and %PVH variation were added in the models. Given that %anterior cement and %posterior cement showed significative collinearity, two separate regression models were developed with these variables.

In the first model, the following variables: were included: %PVH variation and %posterior cement. We first performed an Omnibus test, which showed that the model was statistically significant ( *p* < 0.001). Then, we assessed the Nagelkerke R square, which indicated that the 2 variables in the regression explained 75.1% of the outcome. Of the 2 variables in the analysis, only the %posterior cement showed to be an independent statistically significant predictor of treatment failure (aOR = 1.775; *p* = 0.022). When using the %posterior cement alone, the model was also statistically significant ( *p* < 0.001), the Nagelkerke R square indicated that the variable in the regression explained 74.5% of the outcome. The %posterior cement showed a statistically significant association with surgical failure (aOR = 1.684; *p* = 0.012).

A logistic regression was also performed using %anterior cement, but the model showed a statistically non-significant association with failure.

## Discussion

Vertebral compression fractures are a relatively frequent cause of acute and chronic pain and functional limitations, especially in elderly patients, posing significant impacts on patients' quality of life. Treatment may include conservative measures and, in some cases, surgical procedures, such as a percutaneous technique like kyphoplasty and vertebroplasty. Usually, surgical treatment is opted for when conservative measures fail. However, the decision to undergo surgery must be carefully considered. Therefore, understanding risk factors and identifying possible predictors of surgical failure is essential to guide clinical decision-making and improve patient outcomes. 11

It has been postulated that kyphoplasty results in higher degree of height restoration or kyphotic angle reduction; however, many investigators consider that early clinical results are similar between kyphoplasty and vertebroplasty. 12 For example, Du et al., 3 in a cohort of 112 patients with painful VCFs, compared the clinical and radiological results of kyphoplasty and vertebroplasty and concluded that the clinical results in the first 2 years after surgery are similar between the 2 interventions. In two other works by Liu et al. 13 and Cheng et al., 14 the clinical outcomes between the two groups were similar and, thus, the authors recommended vertebroplasty instead of kyphoplasty due to the higher cost of the kyphotic balloon procedure.

Despite the clinical outcomes being equivalent between kyphoplasty and vertebroplasty, several articles demonstrated that bipedicular kyphoplasty (BKP) provides superior vertebral height restoration, kyphotic angle reduction, and less cement leakage. 3 15 16 Gan et al., 17 in a study involving 38 patients, and Kim et al. 18 with a total of 103 patients with osteoporotic VCFs, demonstrated that BKP had a significant advantage over vertebroplasty in terms of the restoration of the middle vertebral height. Röllinghoff et al. 19 in a prospective study of 90 patients with fresh osteoporotic vertebral fractures concluded that the mean vertebral body height restoration at the 1-year follow-up was significantly higher ( *p* < 0.05) in the kyphoplasty group. Some articles also demonstrated that vertebroplasty was associated with higher rate of complications. 19 20

The current study evaluated the outcomes of percutaneous kyphoplasty in a cohort of 34 patients with vertebral compression fractures. Our results indicated that 58.8% of patients experienced no failure during follow-up, while 41.2% demonstrated surgical failure at varying points. The observed failure rate is noteworthy, with pain recurrence being the most frequent type of failure, occurring in 35.3%. Hackbarth et al. 21 found a similar long-term pain recurrence rate (30.9%) in his study involving 49 patients submitted to kyphoplasty at least 3 months after the intervention. These results emphasize the complexity of managing vertebral compression fractures and the variability in patient outcomes.

The literature indicates several complications linked to kyphoplasty, such as radiculopathies, rib fractures, cement leakage and spinal cord or neural compression. However, our study did not encounter any of those complications. Furthermore, we observed no occurrences of procedure-related infections, cardiac issues, or pneumothoraxes among the patients. 22

In our study, the radiological parameters, AVH, PVH, VKA, and RKA, revealed significant improvements, with increase of vertebral heights and decrease of vertebral kyphotic angles. Several studies have corroborated the effectiveness of kyphoplasty. De Falco et al., 10 using 3 vertebral body height measurements (anterior, middle and posterior), showed in his study of 61 patients submitted to kyphoplasty a good recovery of vertebral heights with a statistically significant increase in all 3 vertebral height measurements posttreatment. The study also showed a wider variation in the anterior height than in the posterior height, which is in line with the results in our study. 10 Lieberman et al., 23 in a study involving 30 patients, found that in 70% of the vertebral bodies, the procedure restored 47% of the lost height. Therefore, substantial height recovery was also reported after kyphoplasty, supporting its effectiveness in treating vertebral compression fractures.

About vertebral height recovery, our investigation revealed some unexpected results that warrant further discussion and should be addressed, as they may significantly influence patient outcomes. When examining the variables between the 2 groups, we found that the %AVH variation was found to be 20.85% in group 1 (without failure) and 12.32% in group 2 (with failure). These findings are in line with the expectation that greater improvements in vertebral body height correlate with better outcomes, despite not being statistically significant. In contrast, the %PVH variations showed a difference of 9.84% in the failure group compared to only 2.36% in the non-failure group, challenging the assumption that increasing vertebral body heights would lead to better outcomes. This raises the hypothesis that an increased variations of the posterior height may lead to worse results on patients submitted to kyphoplasty. Although we could not achieve statistical significance, we believe that the limited sample size poses a significant limitation and further investigation with larger samples may confirm the suspected trend. As far as we know, no references to these hypotheses were found in the literature.

Our study could not find a significant association between kyphoplasty failure and various demographic and clinical variables, including gender, age, time from injury to surgery, and fracture level. This finding contrasts with previous research that suggested that advanced age might impact surgical outcomes. Morozumi et al. 24 showed that older patients had a higher risk of developing adjacent vertebral fractures, with mean ages of 80 and 76 years for affected and non-affected groups, respectively. Nevertheless, our results support the hypothesis that the technical aspects of the procedures may outweigh these demographic variables in determining surgical success.

In the present study, we found a statistically significant association between the percentage of cement in anterior or posterior halves of the vertebrae and treatment failures. Specifically, a higher percentage of posterior cement was an independent predictor of failure. This suggests that increased cement in the posterior region is associated with higher risk of treatment failure. Based on these results, we raised the hypotheses that an inadequate cement placement, especially in the posterior region, compromises the structural integrity of the vertebra, leading to higher risk of future complications. These findings possibly correlate with the hypothesis raised earlier that a larger variation of the posterior height can lead to procedural failure, possibly by increasing local kyphosis.

There are several studies about cement distribution in kyphoplasty; however, to the best of our knowledge, there are not many studies assessing the distribution of cement in anterior and posterior regions. He et al. 25 showed that extensive cement distribution can improve the kyphotic angle and vertebral height successfully, without causing complications. However, in that study, the position of cement within the vertebral body was not discriminated against, making it impossible to correlate with our findings.

Tan et al., 26 in a study involving 137 patients with single-level osteoporotic VCF undergoing percutaneous surgery, divided based on cement distribution in group A (with complete contact of cement with both endplates of the vertebra) and group B (exhibiting partial contact), showed that group A experienced better outcomes in terms of pain relief, vertebral height restoration, and a lower recompression rate. Considering these findings and combining them with the evidence found in our study, the importance of optimal cement distribution during kyphoplasty cannot be overstated, as it has been proven to directly impact patient outcomes. 26

While our study offers valuable insights, several limitations must be acknowledged. Firstly, the research was conducted at a single medical institution and relied on retrospective data, which can introduce concerns regarding data accuracy and generalizability. Secondly, there are some difficulties measuring a tridimensional object such as the body of the vertebrae with 2-dimensional imaging. The vertebral body's height may vary from lateral to medial, which can compromise measurement accuracies. Additionally, the limited sample size constrained the statistical power and generalizability of the findings, potentially affecting their robustness and reliability, making it crucial to interpret them cautiously. Future research with larger cohorts is essential to validate these findings and explore additional factors that may influence surgical outcomes. Finally, there is a scarcity of comparable studies in literature, making it challenging to draw definitive conclusions or conduct more meaningful comparisons.

## Conclusion

In summary, our findings support the evidence that kyphoplasty is a viable and effective options for treating vertebral compression fractures and restoring normal vertebral morphologies. Furthermore, this study identified the percentage of cement distributed in the posterior region of the vertebral body as a significant predictor of surgical failures, suggesting that it may contribute to kyphoplasty failure in patients with VCF.

The identification of specific factors associated with surgical failure, particularly regarding cement distribution, emphasizes the need for meticulous procedural techniques to optimize outcomes. Therefore, we suggest that careful distribution of cements during kyphoplasty, being predominantly placed in the anterior region of the vertebrae, may lead to better outcomes and prevent surgical failure.

Continued research in this area, with larger cohort numbers, is essential for refining patient selection criteria and deeply exploring these suggested results, in order to improve the long-term success rates of kyphoplasty and ultimately leading to better patient outcomes and quality of life.
